# Eruptive Xanthoma With Pancytopenia: A Report of a Rare Case and Diagnostic Challenges

**DOI:** 10.7759/cureus.79737

**Published:** 2025-02-27

**Authors:** Azhar Ahmed, Lujain S Alrohaily, Walaa M Borhan, Fatimah Y Alkhuraisi

**Affiliations:** 1 Dermatology Department, King Fahad Hospital, Medina, SAU; 2 Family Medicine Department, King Salman Bin Abdulaziz Medical City, Medina, SAU; 3 Pathology Department, Taibah University, Medina, SAU

**Keywords:** aplastic anemia, diabetes mellitus, dyslipidemia, eruptive xanthoma, pancytopenia

## Abstract

Eruptive xanthomas (EXs) are yellowish-orange papulonodular cutaneous eruptions typically associated with severe hypertriglyceridemia. We present a case of a 40-year-old female with poorly controlled diabetes mellitus (DM) who developed EX concurrent with pancytopenia. The patient presented with epigastric discomfort, emesis, and generalized cutaneous eruptions. Laboratory findings revealed severe dyslipidemia, pancytopenia, and elevated liver enzymes. Imaging studies indicated interstitial pancreatitis (IP). Despite aggressive multimodal therapy, including immunosuppressive treatments and blood product support, the patient's condition deteriorated rapidly. The pancytopenia persisted, and the patient developed aplastic anemia (AA), ultimately resulting in fatal complications despite aggressive management. This case highlights a potential association between severe dyslipidemia, cutaneous manifestations, and bone marrow dysfunction. Further research is warranted to elucidate the mechanisms linking severe dyslipidemia with hematopoietic dysfunction and to explore potential therapeutic targets that address both the metabolic and hematological aspects of these disorders.

## Introduction

Eruptive xanthomas (EXs), which are cutaneous manifestations of hyperlipidemia, are characterized by the sudden onset of yellowish-orange papules or nodules predominantly on extensor surfaces [1,2]. Although they are frequently associated with lipid metabolic disorders and hypertriglyceridemia, their concurrent occurrence with hematological abnormalities is exceedingly rare and poorly understood [3,4].

Pancytopenia, characterized by reduced counts of all blood cell lines, represents a serious hematological abnormality with significant clinical implications. This unusual association of EX with pancytopenia presents significant diagnostic and therapeutic challenges for clinicians in various specialties. EX occurs when lipid-laden macrophages accumulate in the dermis due to severe hypertriglyceridemia [5]. Previous research has established strong associations between EX and conditions such as diabetes mellitus (DM), obesity, and certain medications [1]. However, the relationship between xanthomas and hematological disorders remains largely unexplored, with only a limited number of case reports suggesting potential correlations [6].

The intricate relationship between hemopoiesis and lipid metabolism has recently been elucidated. In animal models, severe hyperlipidemia affects hematopoietic stem cell activity and the bone marrow microenvironment [7,8]. Emerging research suggests that excessive lipid accumulation may disrupt bone marrow function and hematopoiesis through potential mechanisms, including direct lipid infiltration of bone marrow. Despite recent findings, the mechanisms underlying the co-occurrence of EX and pancytopenia remain unclear. The rarity of such cases has impeded the development of standardized diagnostic protocols and treatment regimens, necessitating clinicians to rely on empirical management approaches [9,10].

This case report aims to address this knowledge gap by presenting a rare case of EX with concurrent pancytopenia in a patient with poorly controlled DM. Through an analysis of the clinical presentation, diagnostic process, and therapeutic course, we endeavored to provide valuable insights into the complex interplay between metabolic disorders, cutaneous manifestations, and hematological abnormalities. This case is significant, as it may elucidate some complex relationships between hematopoiesis and lipid metabolism. We aimed to increase clinicians' awareness of the potential for these uncommon presentations and emphasize the importance of a multidisciplinary approach in patient management by highlighting the diagnostic challenges and therapeutic dilemmas encountered.

## Case presentation

On April 5, 2024, a 40-year-old female Pakistani patient was presented to the emergency department (ED). The patient had a previous diagnosis of DM and was receiving treatment with oral hypoglycemic agents, primarily metformin. Her chief complaints, which persisted for approximately two weeks, included intermittent epigastric discomfort, emesis, and generalized cutaneous eruption.

History

The patient reported that administering analgesics alleviated epigastric discomfort, which was not associated with food intake. The patient denied experiencing pyrexia, anorexia, or unintentional weight loss. Similar episodes had not previously occurred, and the patient did not report any recent medication alterations or contact with individuals exhibiting comparable symptomatology. Due to linguistic barriers, a comprehensive family history of hyperlipidemia, cardiovascular disease, and DM could not be obtained.

Physical examination

**Figure 1:**
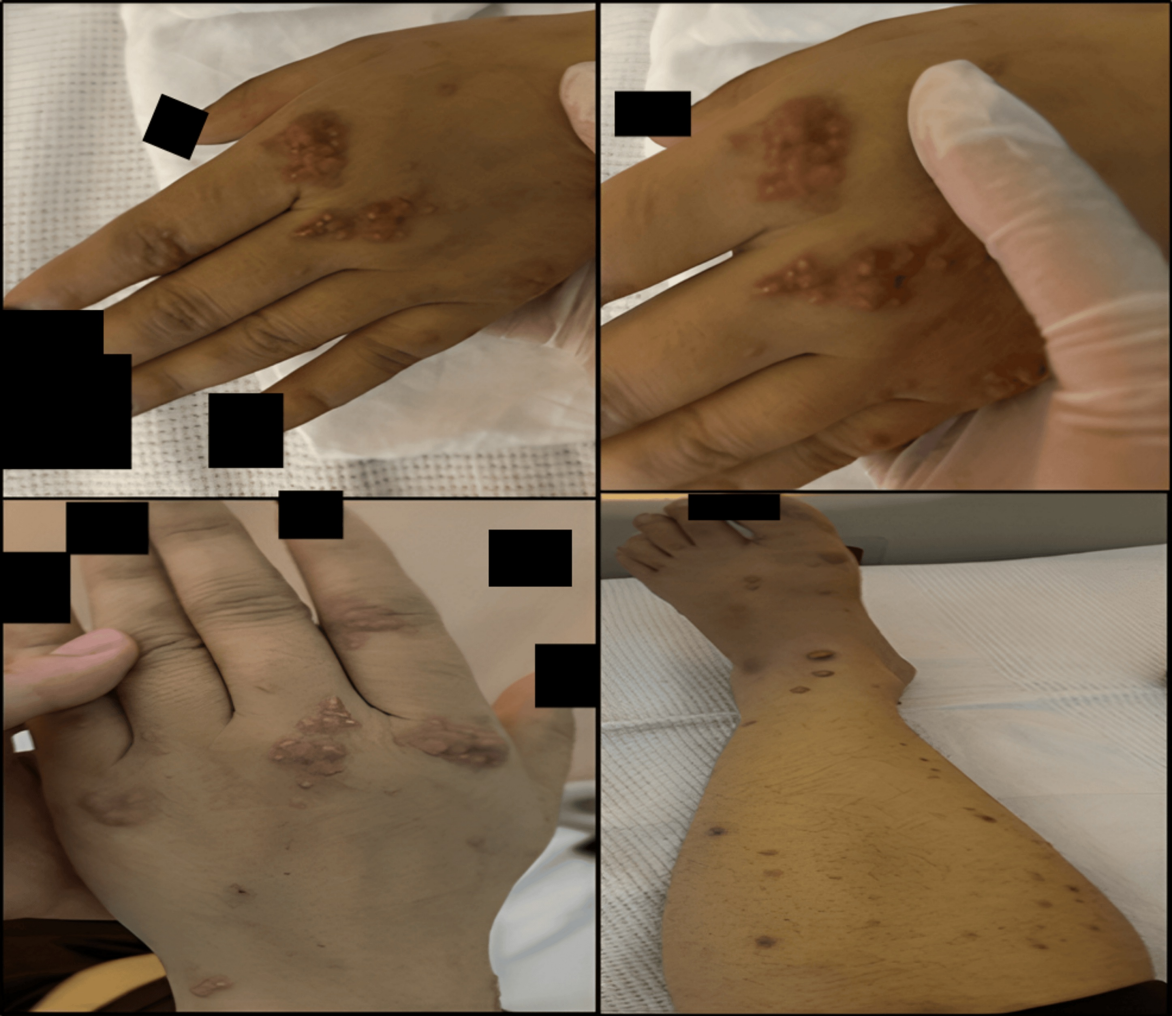
Small, pink-yellow, non-tender papules and nodules coalescing into plaques.

Upon initial examination, the patient appeared healthy; however, generalized jaundice, including scleral icterus, was noted. Abdominal examination revealed a diffusely tender, soft, and lax abdomen, hepatomegaly extending approximately 3 cm below the costal margin, and a negative Murphy's sign. The dermatological assessment revealed a significant non-pruritic, indurated papulonodular rash affecting the extensor and flexor surfaces of the distal upper and lower extremities. The lesions were characterized by small, non-tender, pink-yellow papules and nodules that merged to form plaques covering the extensor surfaces of the arms, forearms, legs, and feet (Figure 1). No additional abnormalities were observed. During the genital examination, the nails, scalp, and oral mucosa appeared unremarkable.

Given these findings, our diagnostic considerations included hyperlipidemia-associated dermatological manifestations, potential hepatobiliary disorders, and unexplained hematological abnormalities. Laboratory investigations were directed toward these differential diagnoses to establish the relationship between the patient's cutaneous manifestations and potential systemic abnormalities.

Initial laboratory findings (April 5, 2024)

Initial laboratory tests revealed significant anomalies across multiple systems. Hemoglobin (Hb) was within the normal range at 12 g/dL; however, the total blood count demonstrated leukopenia with a white blood cell (WBC) count of 2.76 x 10^9/L and thrombocytopenia with platelets at 54 x 10^9/L. The neutrophil and lymphocyte counts were 1.75 x 10^9/L and 0.68 x 10^9/L, respectively. Alkaline phosphatase (ALP) level was 174 U/L and liver function tests indicated significant abnormalities, with markedly elevated aspartate aminotransferase (AST) (1360 U/L), alanine aminotransferase (ALT) (1100 U/L), and gamma-glutamyl transferase (GGT) (1360 U/L) levels. The total bilirubin (358 μmol/L) and conjugated bilirubin (256 μmol/L) levels were also elevated. The lipid profile indicated severe dyslipidemia, with low-density lipoprotein (LDL) at 11.69 mmol/L (450 mg/dL), high-density lipoprotein (HDL) at 0.29 mmol/L (11 mg/dL), total cholesterol at 14.54 mmol/L (562 mg/dL), and triglycerides at 5.83 mmol/L (516 mg/dL). Additional notable findings included elevated lipase level (230 U/L), markedly elevated fasting blood glucose level (441 mg/dL), and poorly controlled diabetes with a glycated hemoglobin (HbA1c) level of 12.6%. Urea, creatinine, thyroid-stimulating hormone (TSH) (0.52 mIU/L), total protein (7.2 g/dL), and other parameters were within normal ranges (Table 1).

**Table 1:** Laboratory investigations in a patient with eruptive xanthoma and pancytopenia from April 5, 2024 AST: Aspartate aminotransferase; ALT: Alanine aminotransferase; GGT: Gamma-glutamyl transferase; LDL: Low-density lipoprotein; HDL: High-density lipoprotein; HbA1c: Glycated hemoglobin; TSH: Thyroid-stimulating hormone; g/dL: Grams per deciliter; mg/dL: Milligrams per deciliter; mmol/L: Millimoles per liter; mIU/L: Milli-international units per liter; U/L: Units per liter; μmol/L: Micromoles per liter

Test	Result	Unit	Reference Range	Status
Hemoglobin	12	g/dL	12-15	Normal
White Blood Cell Count	2.76	x 10^9/L	4-10	Low
Platelet Count	54	x 10^9/L	150-410	Low
Neutrophil Count	1.75	x 10^9/L	2-7	Low
Lymphocyte Count	0.68	x 10^9/L	1-3	Low
Alkaline Phosphatase	174	U/L	40-129	High
AST	1360	U/L	10-50	Critical High
ALT	1100	U/L	0-41	Critical High
GGT	1360	U/L	5-85	High
Total Bilirubin	358	μmol/L	0-20.52	Critical High
Conjugated Bilirubin	256	μmol/L	0-5.13	High
LDL	11.69	mmol/L	0-2.6	High
HDL	0.29	mmol/L	1.03-1.55	Low
Total Cholesterol	14.54	mmol/L	1.3-5.13	High
Triglycerides	5.83	mmol/L	0.34-2.28	High
Lipase	230	U/L	13-60	High
Fasting Blood Glucose	441	mg/dL	70-100	High
HbA1c	12.6	%	4-6	High
Urea	2.6	mmol/L	2.5-8.3	Normal
Creatinine	51	μmol/L	44-115	Normal
TSH	0.52	mIU/L	0.34-5.6	Normal
Total Protein	7.2	g/dL	64-82	Normal

Imaging studies

**Figure 2:**
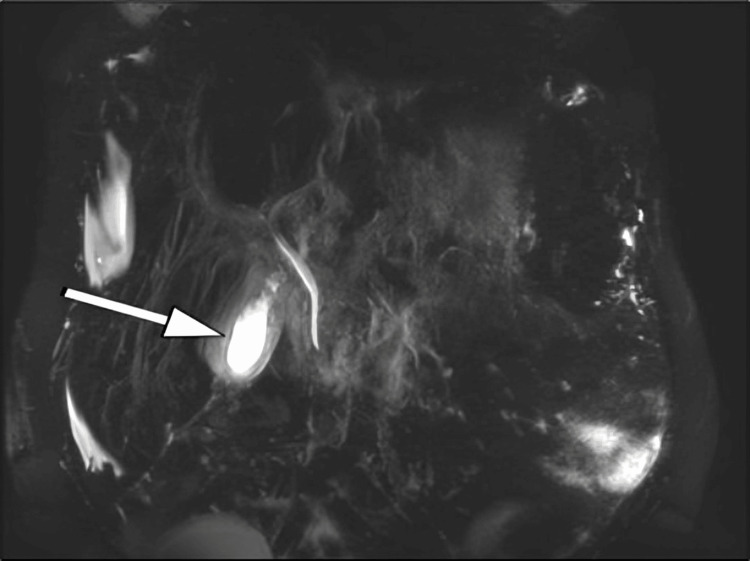
Magnetic resonance cholangiopancreatography (MRCP) image showing an edematous pancreas (white arrow) with surrounding peripancreatic fluid collection, consistent with interstitial pancreatitis.

Further diagnostic imaging was performed to evaluate the patient's condition more comprehensively. On April 5, 2024, abdominal ultrasound indicated the potential presence of a distal common bile duct (CBD) stone and revealed a contracted gallbladder. As the examination was inconclusive regarding acute cholecystitis, a referral for clinical correlation with a magnetic resonance cholangiopancreatography (MRCP) study was initiated. The MRCP revealed an edematous pancreas with peripancreatic fluid, consistent with interstitial pancreatitis (Figure 2).

**Figure 3:**
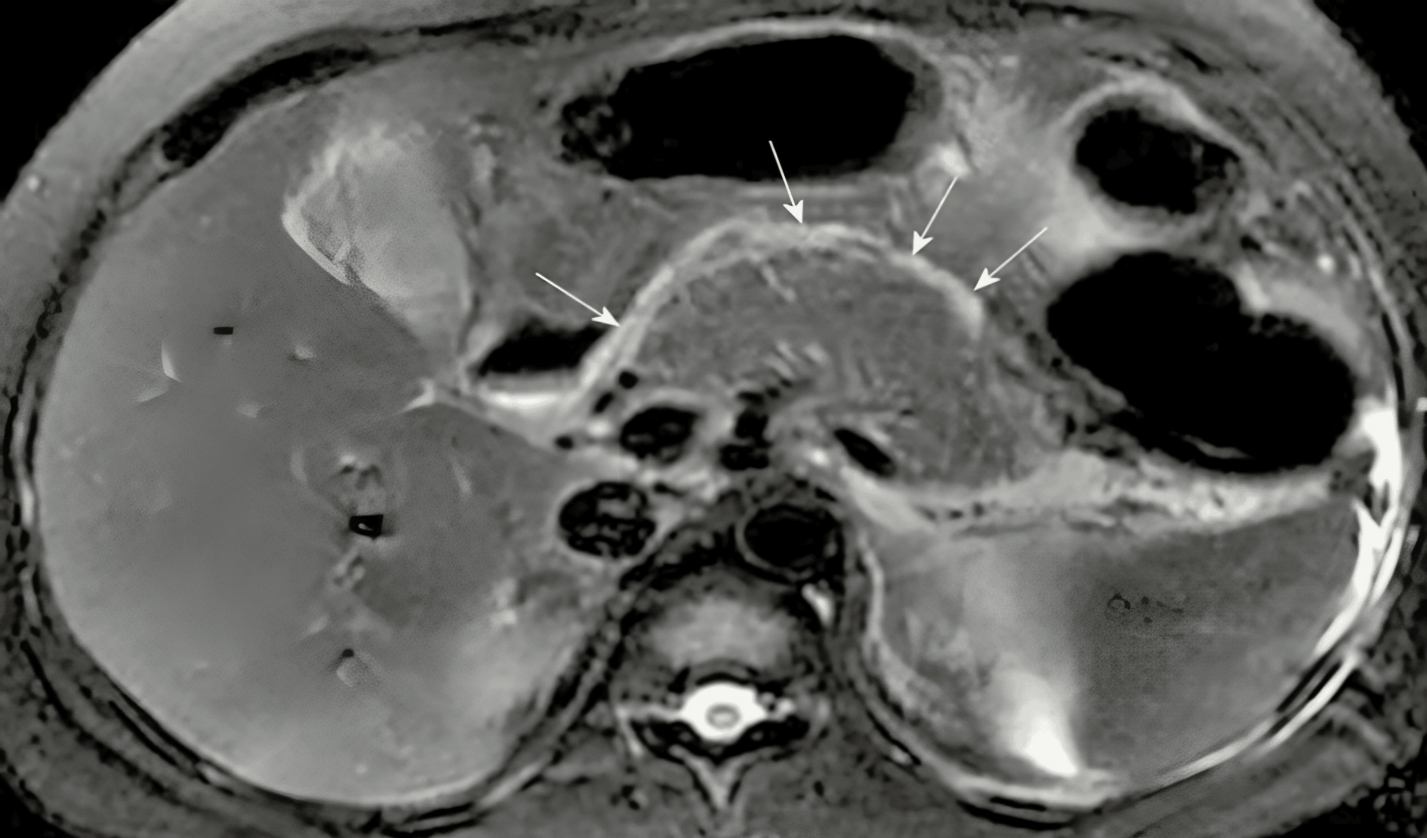
Magnetic resonance cholangiopancreatography (MRCP) image demonstrating a contracted gallbladder with a thickened, edematous wall (0.6 cm) and minimal pericholecystic fluid (white arrows).

Additionally, the MRCP showed a contracted gallbladder with a thick, edematous wall reaching 0.6 cm in thickness, along with minimal pericholecystic fluid (Figure 3). The radiologist noted that these findings might indicate a more complex medical condition, underscoring the intricacy of the patient's case.

Histopathology

**Figure 4:**
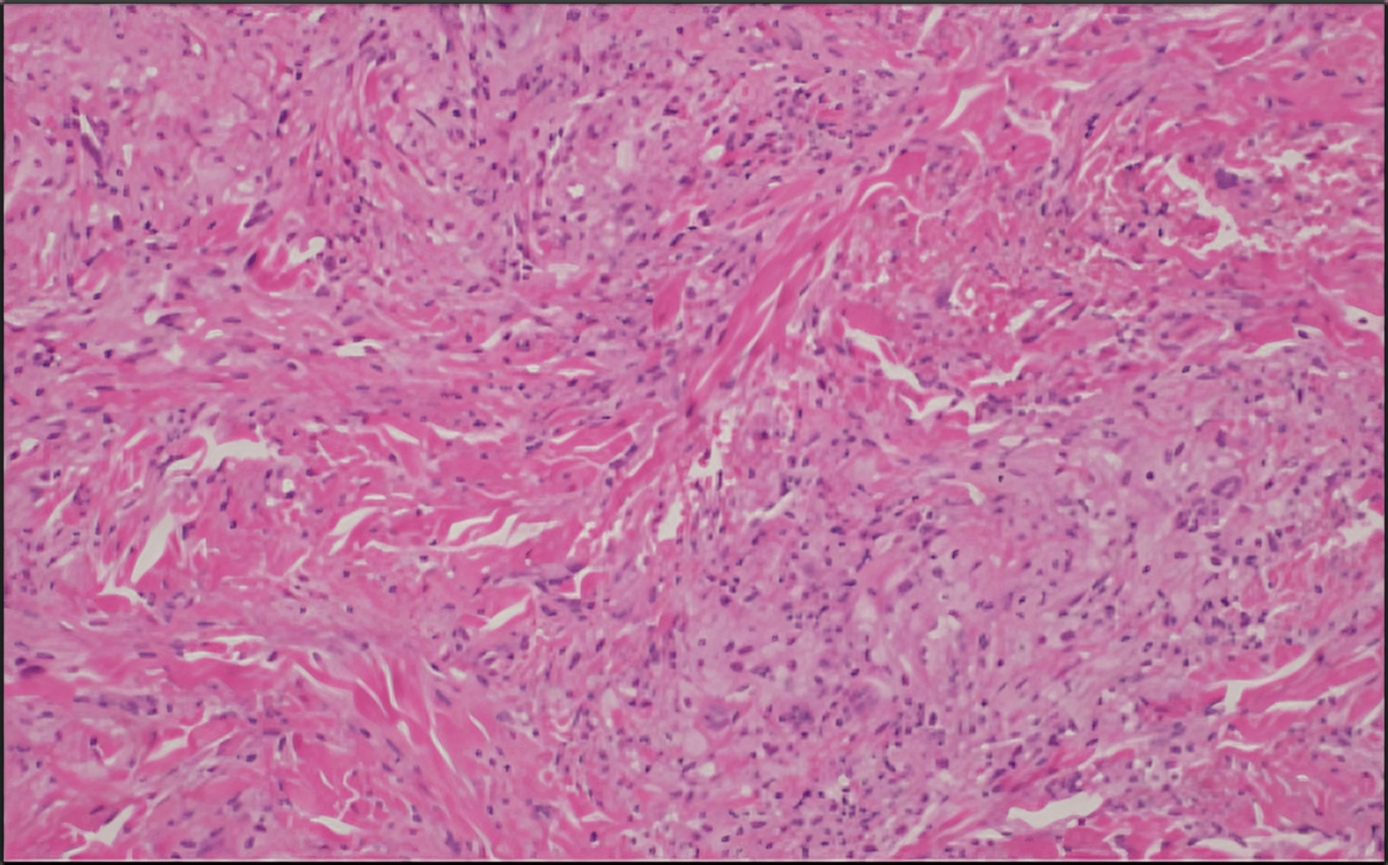
Histopathological image showing a dense population of foamy histiocytic infiltrates in the dermis.

Histopathological examination of a lesion biopsy confirmed the clinical diagnosis and provided insight into the pathological mechanisms underlying the patient's presentation. The histological sections revealed a dense dermal infiltrate composed predominantly of foamy histiocytes and lipid-laden macrophages. These cells were dispersed throughout the dermis, forming clusters interspersed with areas of lipid deposition, as visualized under high-power magnification (Figure 4).

**Figure 5:**
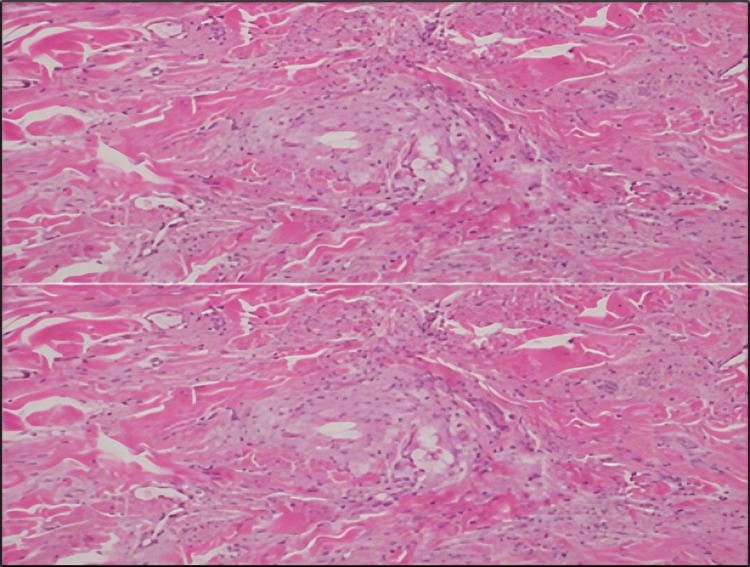
High-power magnification showing a dermal foamy histiocytic infiltrate.

The infiltrate exhibited a characteristic foamy appearance due to the accumulation of intracellular lipids, a hallmark feature of EX. No multinucleated giant cells, granulomas, or other signs of chronic inflammation were observed, excluding other possible differential diagnoses such as xanthogranulomas or infectious etiologies (Figure 5).

Histopathological sections reveal a dermal foamy histiocytic infiltrate with lipid deposition. At high power, the infiltrates consist of foamy histiocytes with focal lipid accumulation, while giant cell formations are absent. The morphological finding, along with the clinical impression, supported the diagnosis of EX. These findings, along with the patient's severe dyslipidemia, confirmed the diagnosis of EX. The absence of necrosis or atypical cells confirmed its benign nature. This histopathological evaluation not only provided definitive evidence for the cutaneous manifestation but also emphasized the systemic impact of the underlying metabolic disturbance. The results highlighted the direct relationship between extreme lipid dysregulation and the deposition of lipids in dermal histiocytes, offering valuable insights into the pathophysiology of this rare dermatological condition.

Clinical course and management

Initial Management (April 5-8, 2024)

The patient's clinical course was complex and rapidly evolving. The initial treatment regimen comprised empirical antibiotic therapy for one week, consisting of ceftriaxone 2 g intravenous (IV) once daily and metronidazole 1 g IV three times daily. Heparin was administered for two days, and insulin glargine was initiated for glycemic control.

Progressive Pancytopenia and Hematologic Support (April 9-15, 2024)

On April 9, the patient developed significant thrombocytopenia (platelet count decreased to 16 × 10^9/L). This necessitated platelet transfusions (six units between April 9 and 15) and the initiation of tranexamic acid 1000 mg three times a day for three days. On April 15, further investigations revealed a positive blood culture for *Brucella*, prompting the commencement of piperacillin-tazobactam treatment. Additional therapeutic interventions included the initiation of filgrastim 300 mg subcutaneously once daily, bone marrow biopsy, IV immunoglobulin (IVIg) 6 g IV daily for two days, dexamethasone 40 mg in 100 milliliters (ml) normal saline, and transfusion of two units of packed red blood cells (pRBC).

Further Complications and Interventions (April 16-23, 2024)

Herpes simplex virus polymerase chain reaction (HSV PCR) testing, dexamethasone, and ongoing blood product support were integral to continued management. IV paracetamol was administered to manage a fever spike of 38.8°C on April 23.

Terminal Phase and Outcomes (April 24-May 2, 2024)

The later stage of care was characterized by daily platelet transfusions, continuous blood product support, cyclosporine initiation, and persistent pyrexia. Topical betamethasone was prescribed on May 1, following a dermatological consultation. On May 2, the patient was diagnosed with aplastic anemia (AA) and transferred to the intensive care unit. The terminal phase was marked by severe malnutrition, diarrhea, significant weight loss (>30 kg over two weeks), and persistent fever. Despite intensive care, the patient's condition deteriorated, ultimately resulting in mortality. The patient's death primarily resulted from progressive bone marrow failure and subsequent AA, complicated by febrile neutropenia and sepsis, despite aggressive multimodal therapy.

Key clinical observations

**Figure 6:**
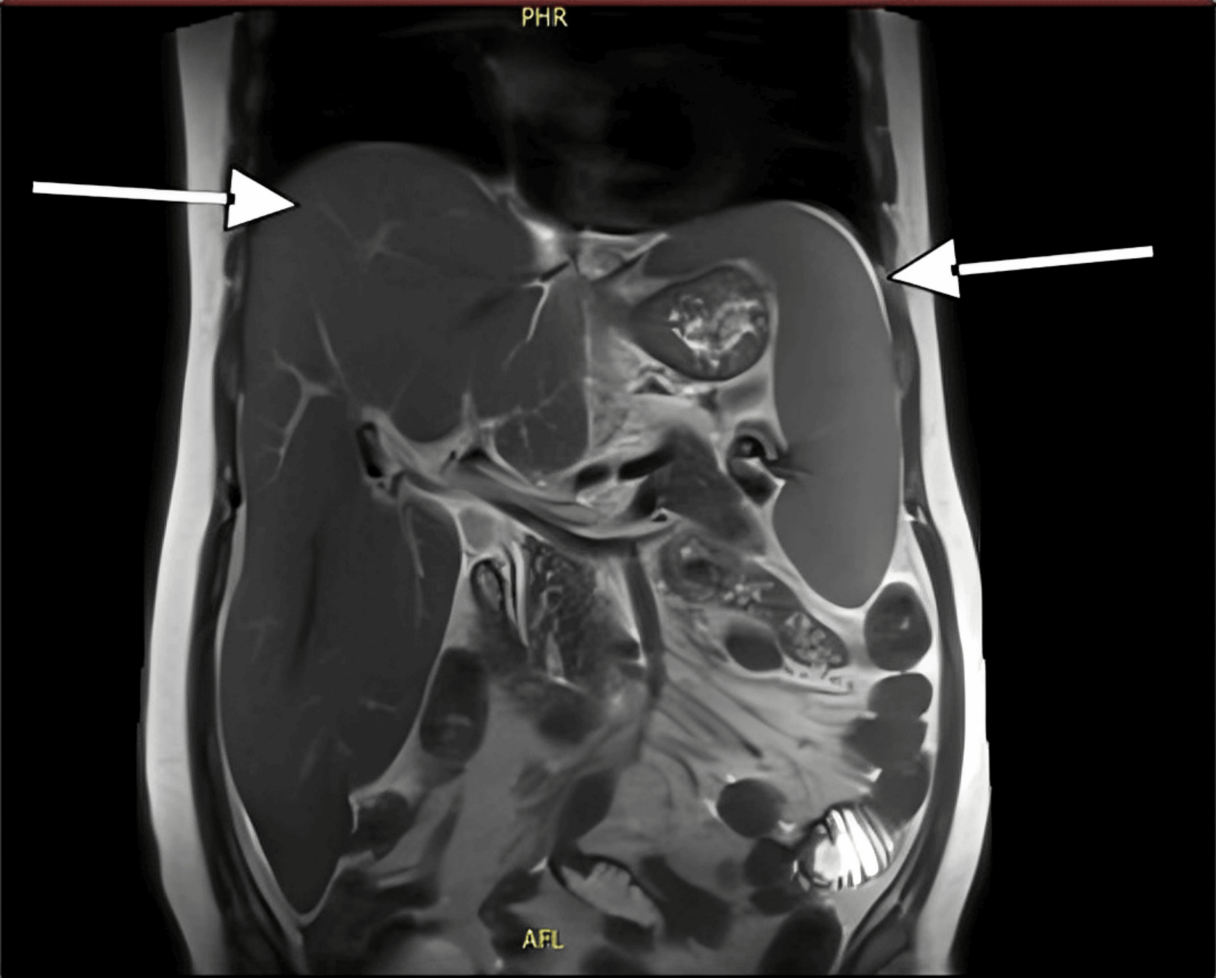
Imaging showing hepatosplenomegaly (white arrows).

Several significant observations were made during the patient's clinical course. Hepatosplenomegaly progressed, with the spleen becoming palpable 4 cm below the costal edge and the liver enlarging to 10 cm below it. This was evident in imaging studies confirming hepatosplenomegaly (Figure 6). Following administering IVIg and 100 mg IV hydrocortisone, a marked improvement was observed in the lipid profile, liver function tests, and skin lesions. Despite multiple therapeutic interventions, including IVIg, filgrastim, cyclosporine, cyclophosphamide, eltrombopag, vitamin K, tranexamic acid, and recurring blood and RBC transfusions, pancytopenia persisted. This case report illustrates the patient's complex and rapidly progressing condition, characterized by EX, severe hyperlipidemia, pancytopenia, and multiple organ involvement. This case presented significant diagnostic and therapeutic challenges that ultimately resulted in poor prognosis despite aggressive multimodal therapy.

## Discussion

This case report presents a patient with poorly managed DM who developed EX concurrent with pancytopenia, an uncommon and complex clinical condition. This case provides several significant insights and raises intriguing questions regarding the interrelationship between cutaneous manifestations, hematological function, and lipid metabolism. Previous reports have indicated that the initial presentation of EX is consistent with severe hypertriglyceridemia [1,2]. However, this case deviates from the typical presentation of EX due to the simultaneous occurrence of pancytopenia. Although cutaneous manifestations of systemic disorders are well documented [4,11], the specific association between EX and hematological abnormalities is infrequently reported in the literature.

The patient's severe dyslipidemia, characterized by markedly elevated total cholesterol (14.54 mmol/L) and triglycerides (5.83 mmol/L) levels, aligns with the pathophysiological mechanism of xanthoma formation. Zak et al. posited that severe hypertriglyceridemia frequently results in the accumulation of lipid-laden macrophages in the dermis, leading to the development of xanthomas [5]. The rapid improvement in skin lesions following IVIg and hydrocortisone therapy supports the hypothesis that addressing the underlying metabolic disturbances may resolve cutaneous manifestations.

The potential mechanisms linking severe dyslipidemia with bone marrow dysfunction deserve further exploration. Possible pathways include (1) direct lipid-induced suppression of hematopoietic stem cells, (2) dyslipidemia-triggered inflammatory cytokines affecting hematopoiesis, and (3) lipid infiltration of the bone marrow microenvironment. Recent studies by Rasheed et al. have suggested that hyperlipidemia may influence hematopoiesis through its effects on the splenic niche [7]. Lee et al. elucidated the complex relationship between hematopoietic stem cell function and lipid metabolism [8]. This case contributes to the growing body of evidence indicating a potential link between bone marrow dysfunction and severe dyslipidemia.

The patient's poorly controlled diabetes (HbA1c 12.6%) likely contributed to both dyslipidemia and EX development. This observation aligns with the findings of Shrestha et al., who reported a case of EX as a warning sign of uncontrolled hypertriglyceridemia in a patient with poorly controlled type II DM [6]. However, in this case, pancytopenia introduced a complexity not typically encountered in such presentations.

The patient management presented challenges as pancytopenia persisted despite multiple therapeutic interventions, including IVIg, filgrastim, cyclosporine, and eltrombopag. This therapeutic resistance suggests a potential disruption of hematological function that may be more extensive than that anticipated from metabolic derangement alone. Progression to AA raises questions regarding possible underlying genetic or autoimmune factors that may have contributed to the patient's condition.

Notably, the significant improvements in lipid profile, liver function tests, and skin lesions following IVIg and hydrocortisone treatment are of interest. This response suggests a potential immunological component of the patient's illness, consistent with our evolving understanding of inflammation's role in hematological dysfunction and metabolic disorders [7]. The development of hepatosplenomegaly throughout treatment further underscores the systemic nature of the condition. This manifestation bears resemblance to lysosomal storage diseases, although the acute onset and rapid progression observed in this case are atypical for disorders such as pancytopenia and dyslipidemia.

The patient's subsequent deterioration and onset of febrile neutropenia emphasize the severity and potential lethality of this constellation of symptoms. This outcome underscores the necessity for aggressive case management and early identification of such complex cases. Our case exemplifies the extensive ramifications of aberrant lipid metabolism, building upon the seminal work of Brown and Goldstein on cholesterol homeostasis [9]. This case also highlights the current gaps in understanding the interplay between metabolic disorders and hematological function.

This case study has several limitations. As a single instance, it cannot establish causality or determine the prevalence of a specific constellation of symptoms. Moreover, the rapid progression of the patient's condition precluded more comprehensive investigations to elucidate the underlying pathophysiology.

## Conclusions

The current case of EX with pancytopenia represents a novel clinical entity that challenges our understanding of the relationship between lipid metabolism, cutaneous manifestations, and hematological function. This case emphasizes the importance of early recognition of severe dyslipidemia's potential systemic effects beyond cardiovascular complications. Clinicians managing patients with EX should consider hematological evaluation, especially in cases with poorly controlled metabolic disorders. This underscores the importance of considering atypical correlations when evaluating patients presenting with EX and highlights the necessity of a multidisciplinary approach in managing such complex cases. Future research should focus on elucidating the mechanisms linking severe dyslipidemia with hematopoietic dysfunction, particularly investigating immunological mechanisms potentially connecting lipid metabolism with hematopoietic function, and exploring genetic factors that might predispose certain individuals to develop bone marrow dysfunction in the setting of severe dyslipidemia.

Early multidisciplinary management involving dermatology, hematology, and endocrinology specialists may improve outcomes in such complex cases. Further studies are needed to develop potential therapeutic targets that may address both the metabolic and hematological aspects of these disorders, potentially preventing the progression to severe complications as observed in this case.
